# Supplementary material for: Air Passenger Travel and International Surveillance Data Predict Spatiotemporal Variation in Measles Importations to the United States
Source: Pathogens. 2021 Feb 3;10(2):155. doi: 10.3390/pathogens10020155 (PMC7913265; doi:10.3390/pathogens10020155)
Supplement: Supplementary file 1 [file pathogens-10-00155-s001.pdf]

Supplement

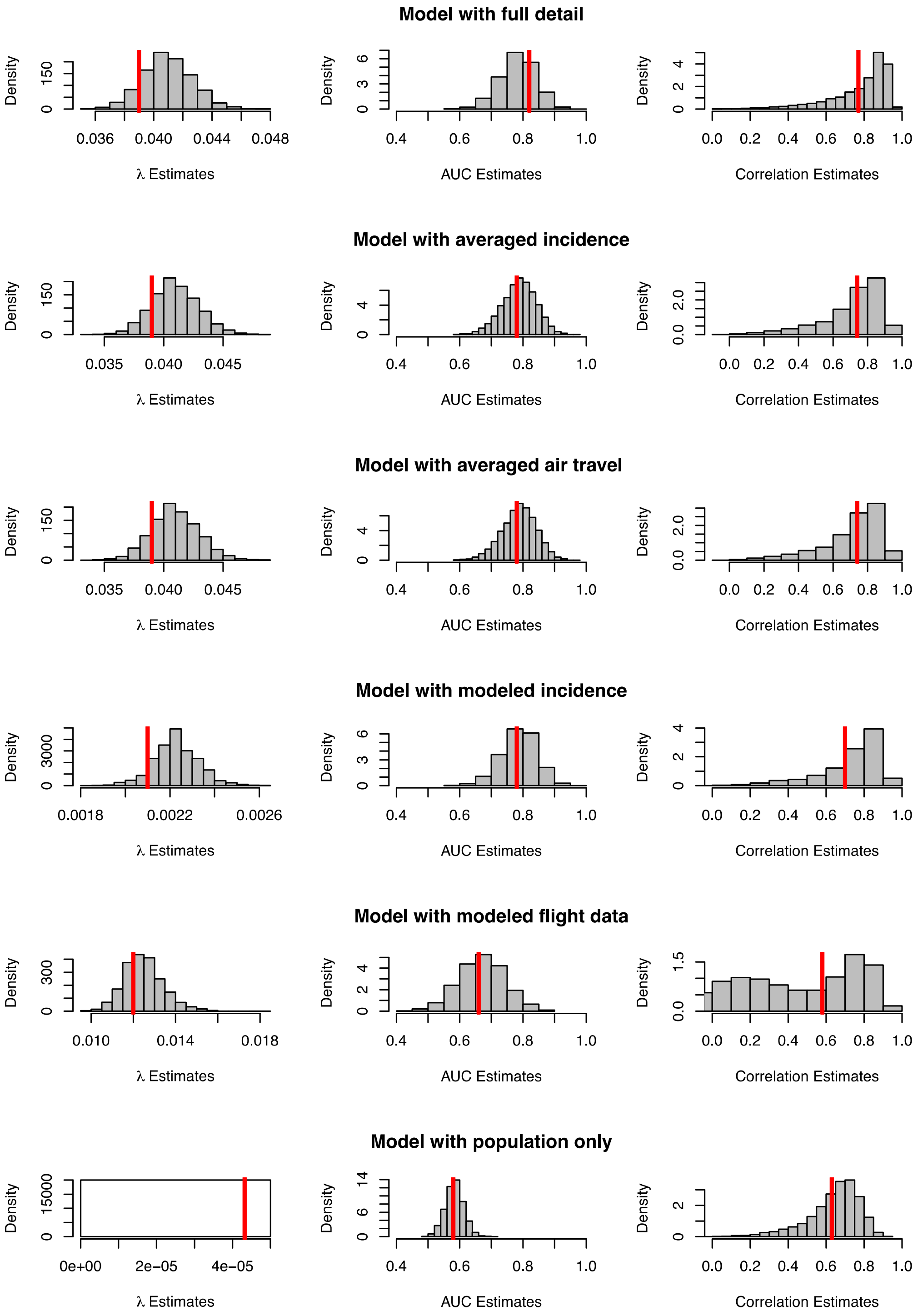

Figure S1. Histograms of partial fit model statistics. Values generated when each model was fit to the complete dataset are shown in red. The null model's resampling process consistently generated identical λ, likely a result of the large population values in question, and as a result no histogram is shown.
